# Root Canal Morphology of Mandibular Canine in an Iranian Population: A CBCT Assessment 

**DOI:** 10.22037/iej.2017.16

**Published:** 2017

**Authors:** Ali Soleymani, Nafiseh Namaryan, Ehsan Moudi, Ali Gholinia

**Affiliations:** a *Department of Dentistry, Babol University of Medical Sciences, Babol, Iran*

**Keywords:** Canine, Cone-Beam Computed Tomography, Root Canals

## Abstract

**Introduction:**

The present study was conducted to assess the morphology of mandibular canines using cone-beam computed tomography (CBCT) in a north Iranian population.

**Methods and Materials:**

For the morphological assessment of mandibular canines, 150 CBCT images taken from patients for different reasons were used. The mandibular canines were examined in sagittal, coronal and axial dimensions. The canal pattern, number of roots/canals, the tooth length, the orientation of the roots and the position of the apical foramina were evaluated and the effect of gender on each variable was assessed. The obtained data were analyzed using the Chi-square and student’s t-tests.

**Results:**

According to the Vertucci’s criteria, the most common pattern was type I morphology (89.7%), followed by types III (5.7%), II (3.7%) and V (1%). No significant differences were observed between the male and female patients in terms of canal type (*P*>0.05). Gender difference is a factor which affected the root length and the number of mandibular canine root and root canal. There were 296 single-root and four double-root canines. The double-root canines and mandibular canine with two canals were significantly more common among men than women (*P*=0.00). The apical foramen was laterally positioned in 68.3% and centrally in 31.7% of the cases, and the root curvatures were mostly oriented toward the buccal region. No significant statistical difference was observed for mentioned parameters in right and left half of the jaw.

**Conclusion:**

Due the diverse morphology and the potential presence of a second mandibular canine among Iranians, dentists should perform endodontic treatments with greater care. CBCT is an accurate tool for the morphological assessment of root canals.

## Introduction

The main objectives in endodontics include the biomechanical cleaning of the pulp cavity and root canal and preparation of this space for receiving the filling material and the complete hermetic sealing of the apical and coronal regions [1, 2]. Having proper knowledge about dental morphology and being able to make an accurate interpretation of it and having adequate access to its sources are prerequisites of a successful root canal treatment and determination the treatment outcome [-].

Canines are known as the cornerstone of the dental arch. Both upper and lower canines in the labial part possess aesthetic value and help create natural face shapes apart from their functional role as occlusion guidance [6]. 

The mandibular canine is usually single-rooted; and according to numerous studies, 1.7 to 6.2% of these teeth are double-rooted and 10.6% have two or more canals [1]. Pécora *et al.* [7] found that 98.3% of the canines were single-rooted, 93.3% of which then had single canals, 4.9% had double canals and a foramen and 1.2% had double canals with double foramina. Vaziri *et al.* [8] studied the anatomy of mandibular canines in an Iranian population and found that 88% of the canines were single-rooted and 12% were double-rooted.

Proper anatomical knowledge is essential in the treatment of canine roots in different cases [9]. The morphological study of root canals can be performed through different ways, including staining and tooth clearing, tooth sectioning, conventional radiography, digital radiography and conventional computed tomography (CT) [9, 10]. The ideal technique is the one that is accurate, simple, non-invasive and capable of *in vivo* application [9]. Cone-beam computed tomography (CBCT) has become a successful tool to explore the root canal anatomy [11, 12]. Neelakantan *et al.* [13] had concluded that CBCT is an accurate as modified canal staining and clearing technique which is a gold standard in identifying root canal anatomy. The main benefits of this technique include producing three-dimensional images compared to conventional radiography, being non-invasive, reducing superimpositions in intra oral and extra oral anatomies and their surrounding structures and lower radiation doses and costs compared to conventional CT [-]. Many studies have noted the effect of ethnicity on the anatomic diversity of root canals, and since studies on morphological assessment of mandibular canines on Iranian population using CBCT are limited, the present study was conducted to investigate the morphological diversity of mandibular canines using CBCT in a north Iranian population.

## Materials and Methods

The present study evaluated the three dimensional CBCT images of 300 completely erupted mandibular canines belonging to 150 patients with a mean age of 42.5 years that presented to a private radiology clinic in Babol, Iran, over a one-year period. Only the canines with no endodontic treatments were examined. CBCT images that fulfilled the following criteria were selected: high-quality CBCT images that showed mandibular canines with completely erupted roots, untreated root canals, absence of coronal or post-coronal restorations, absence of periapical lesions and root resorptions and bilateral presence of mandibular canines.

All the CBCT images were taken with New Tom 5G (QR SRL Co., Verona, Italy) at 90 kVp, 41.05 mA and an exposure time of 9.0 sec and 8×8 FOV. The voxel size of the images was 75 μm. The CBCT cross-sections were 1 mm thick taken from the apical to the coronal regions. The canines were categorized by the patients’ gender, tooth quadrant (left or right), the number of the roots and canals and the root canal morphology.

The OnDemand 3D software (Cybermed Inc, Irvine, CA) was used to analyze the CBCT images. Each canine was evaluated in three planes, including axial, sagittal and coronal planes (Figure 1).

The anatomic length of the root was measured in sagittal and axial planes from the CEJ to the apex. The position of each apical foramen was classified as central (at the tip of the root apex) or lateral (away from the tip of the root apex or off-centered). The root curvatures were evaluated in three plans. The inter orifice distance between two canals or two roots was measured from the edge of one canal to the other in the axial plane using a ruler software. The prevalence of each Vertucci type was determined through evaluating the sagittal and axial planes.

The following information was recorded and analyzed: The root canal pattern, the number of canals and roots for each canine, the possibility of morphological bilateral symmetry, the foramen and root curvature positions, the distance between two root canal orifices of the mandibular canines with two root canals and the anatomical length of the root.

The data were analyzed in SPSS software (SPSS version 20.0, SPSS, Chicago, IL, USA) using the Chi-squared test and the t-test. The level of statistical significance was set at 0.05.

## Results

Totally 86 of the samples were female and 64 were male. Table 1 presents the canal configurations according to the Vertucci’s criteria. The most common detected pattern was type I morphology (89.7%), followed by type III (5.7%) and type II (3.7%). No significant differences were observed between the male and female patients regarding this prevalence (*P*>0.05) (Table 1).

A total of 265 (88.33%) of the canines had one root and one canal while 35 (11.66%) had two canals (four canines with two roots and 31 canines with one root and two canals). The prevalence of having a mandibular canine with two canals was higher in the men than in the women. 

The mean anatomical length of the roots was 15.58 mm. The root length was significantly higher in men (*P*=0.00) (Table 2). The apical foramen was positioned laterally and centrally in 68.3 and 31.7% of the cases, respectively. No significant differences were observed between men and the women (*P*>0.05); 60% of canines had no root curvature while 11.7% had buccal and 8.7% distal orientations. The root curvatures were mostly oriented toward the buccal region (11.7%). No significant differences were observed between men and women in this regard (*P*>0.05).

The mean distance between the two orifices was 1.28 mm (SD=0.22). No significant differences were observed between the right and left canines regarding any of the parameters evaluated in the study (*P*>0.05). The probability of morphological bilateral symmetry for the type of root canal of mandibular canine was 95.4%. 

## Discussion

The present study found the most common root canal morphology for mandibular canines to be type I (89.7%), as consistent with the results obtained by Pineda and Kuttler (81.5%) [18], Rahimi *et al.* (91.6%) [19], and Pecora *et al. *(92.2%) [7]. Of all the studies conducted on mandibular canines, Pecora *et al.* [7] reported the highest prevalence of type I morphology (92.2%). The second most common morphology detected in the present study was type III (5.7%), followed by type II (3.7%) and type V (1%). In the study by Vertucci [20], the second and third most common morphologies detected were type II (14%) and type III (2%), respectively. The prevalence of type IV morphology for mandibular canines was reported to be 6% by Vertucci [20], 5% by Pineda *et al*. [18] and 1.2% by Pecora *et al.* [7]; however, the present study detected no cases of type IV morphology in mandibular canine in north Iranian population. The present study found that 11.6% of the cases had a second canal, which is consistent with the numbers obtained by Vaziri *et al.* (12%) [9], Green (13%) [21], Hessions (11%) [22], Kaffe *et al.* (13.75%) [23] and Rahimi (12.08%) [19], but higher than the numbers obtained by Bellizzi and Hartwel (4.11%) [24], and Ingle *et al.* (6%) [25], and lower than those obtained by Caliskan *et al.* (19.5%) [26], Vertucci (22%) [20], Sobhani *et al*. (28.2%) [27] and Sert *et al*. (24%) [28]. 

**Table 1:** Root canal pattern in mandibular canine

**Type**	**Total (300)**	**Left (150)**	**Right (150)**	**Men (128)**	**Women (172)**
**I**	89.7 (269)	90 (135)	89.3 (134)	38.7 (116)	51.0 (153)
**II**	3.7 (11)	2.7 (4)	4.7 (7)	2.3 (7)	1.3 (4)
**III**	5.7 (17)	6.0 (9)	5.3 (8)	1.7 (5)	4.0 (12)
**V**	1 (3)	1.3 (2)	0.7 (1)	0.0 (0)	1.0 (3)

The present study found the prevalence of double-rooted canines as 1.33%, which is consistent with the results obtained in previous studies (0.3% to 6.2%) [7, 27]. Lambrianidis *et al.* [29] argued that the disparity in the results of morphological studies may be due to the differences in the used classification systems, the sample size and the racial differences. Vertucci *et al.* [30] and Amin Sobhani *et al. *[27] reported the most prevalent root curvature in mandibular canines as straight, followed by distal and labial curvatures. The present study found that 60% of mandibular canines were straight, while 11.7% had buccal and 8.7% distal orientations and the least prevalent root curvature in mandibular canines in our study oriented toward the mesio lingual region. Furthermore, the apical foramina were positioned laterally in 68.3% of the cases and centrally in 31.7%. Previous studies have reported similar results in different populations [7, 25, 28, 31]. The present investigation of an Iranian population revealed the majority of the apical foramina in mandibular canines to be laterally positioned. Sufficient care should therefore be taken in determining the duration of functioning, clearing and shaping of mandibular canines. Sert and Bayirli [28] consider gender as an important factor for assessing root canal morphology before treatment. In our study, gender affected root length and also the number of mandibular canine canals. In the present study, the average length of mandibular canine root was 15.51 mm and the root was significantly longer in men than in women and these results were consistent with the studies by Versiani *et al*. [31] and Amardep *et al.* [6]. In addition, the prevalence of two canals was more in men than in women, which is consistent with the findings of Sert and Bayirli [28] and altosny *et al*. [32]. But, Kayaglu *et al.* [33] reported that canines with two canals are often more in women than in men. Our findings about the variations of mandibular canine canal are rather different from the results of previous results in other populations of Iran and other races. The results of our study about the type of tooth are different from the studies of Pecora *et al.* [7], Pineda and Kuttler [18] and Vertucci [20] and about the number of canals and mandibular canine root are different from the studies of Vertucci [20], Caliskan *et al.* [26] and Ingle *et al.* [25] and the differences can be attributed to race as an important factor. In our study and other studies in the past, no statistically significant differences were observed between right and left half of the jaw and canine root canal morphology. 

In clinical terms, morphological bilateral symmetry is crucial in the treatment of patients with contra lateral teeth [35]. The present study found the probability of morphological bilateral symmetry in mandibular canines to be 95.4%; this finding helps dentists better predict the morphology of mandibular canines in complex cases. Furthermore, the results obtained in the present study regarding mandibular canine canal diversity are somewhat different from those obtained in previous studies conducted in Iran [9, 27] or on other races. These differences can be explained by ethnical differences as well as the differences in other parameters (such as the study methods, classification system and sample size) [29, 32]. Prior to this study, no studies had addressed the anatomic diversity of mandibular canines in northern Iranian population but the results of this study cannot be generalized to the whole population of northern Iran as the sampling was conducted in a specific center in northern Iran; thus it is suggested that further studies be conducted in different parts of northern Iran to obtain more accurate results. 

## Conclusion

In this study, 1.33% of mandibular canines were double-rooted and 11.6% had double canals. These findings emphasize the importance of clinician’s knowledge of morphological diversity of root canals. Since leaving a canal untreated is one of the main causes of root canal treatment failure, the presence of a second canal must always be considered by the dentist in mandibular canine root canal treatments. Cone-beam computed tomography provides an accurate tool for the morphological assessment of canines.

**Table 2:** Anatomic length of root of mandibular canine

Total (*n*=300)	Women (*n*=172)	Men (*n*=128)	Left (*n*=150)	Right (*n*=150)	Anatomic length
**12.2**	12.2	13.1	12.2	12.9	**Minimum**
**18.8**	18.8	18.6	18.8	18.6	**Maximum**
**15.58 (1.27)**	15.13 (1.06)	16.19 (1.29)	15.61 (1.30)	15.55 (1.25)	**Mean (SD)**
